# Supplementary material for: Dose Reduction to Motor Structures in Adjuvant Fractionated Stereotactic Radiotherapy of Brain Metastases: nTMS-Derived DTI-Based Motor Fiber Tracking in Treatment Planning
Source: Cancers (Basel). 2022 Dec 31;15(1):282. doi: 10.3390/cancers15010282 (PMC9818359; doi:10.3390/cancers15010282)
Supplement: Supplementary file 1 [file cancers-15-00282-s001.zip › cancers-2053448-supplementary.pdf]

cancers

MDPI

6. Supplemental Data

Dosimetric analysis and comparative evaluation

Gradient indices (GI) were applied to evaluate the dose gradient outside of the PTV

IVa. $GI_{high} = \frac{IV50\%}{IV90\%}$ [39,42], presenting the dose gradient from the high to middle dose.

IVb. $GI_{low} = \frac{IV25\%}{IV50\%}$ [39,42], representing the dose-fall-off from the middle to the low dose.

IV25%, IV50%, and IV90% are the isodose volumes covering 25%, 50%, and 90% of the prescription dose, respectively. Higher values represent a steeper dose gradient.

Results

With plan adaption to avoid $FT_{mot.TMS}$ the gradient index for high isodose-levels was slightly more ideal (p = 0.02), the gradient index for low idose-levels was inferior compared to regular plans (p < 0.001).

Table S1. High and low gradient indices in regular treatment plans (regular) and with sparing (spare) DTI-FT based CST. Gradient indices changes are minor, even though significant.

PTV	regulare	DTI-FT spared	*p*-Value
**Gradient-Index high**	2.7 ± 0.3	2.8 ± 0.4	$p = 0.02$
**Gradient-Index low**	2.9 ± 0.5	2.6 ± 0.5	$p < 0.001$

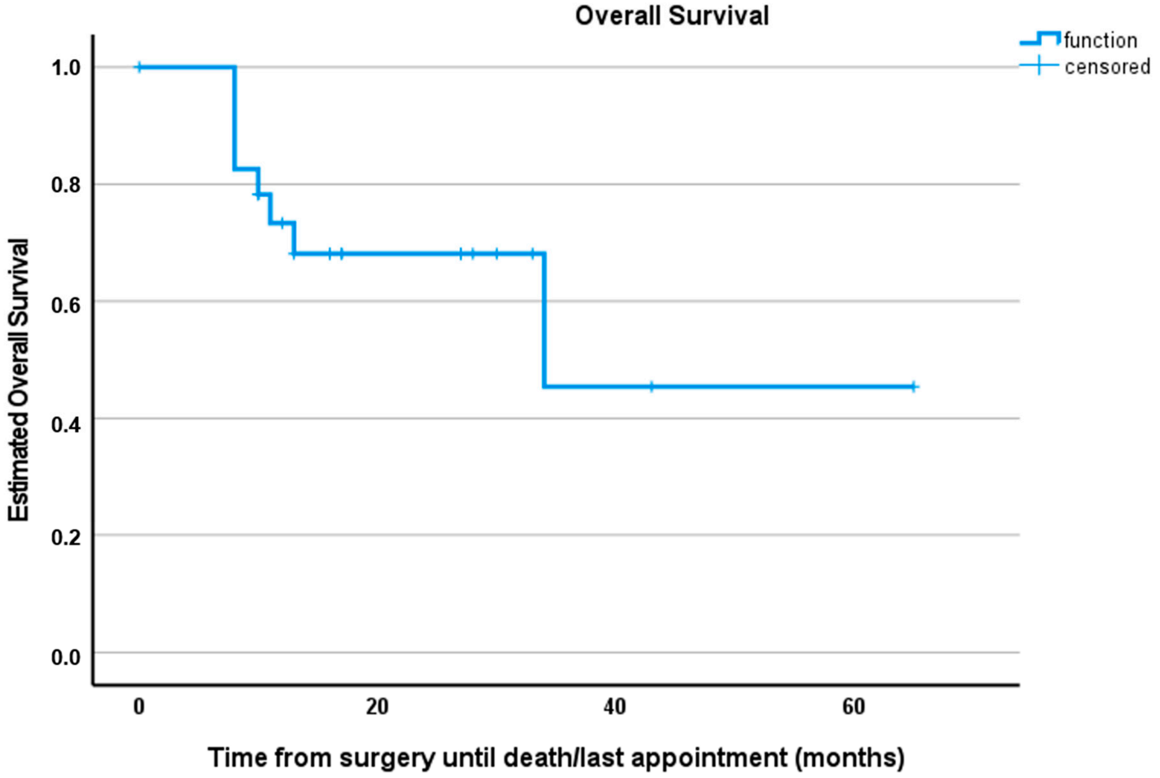

Figure S1. Kaplan-Meyer curve for overall survival. Estimated OS at twelve months was 68.1% (95%-CI 48.5 – 87.7%).

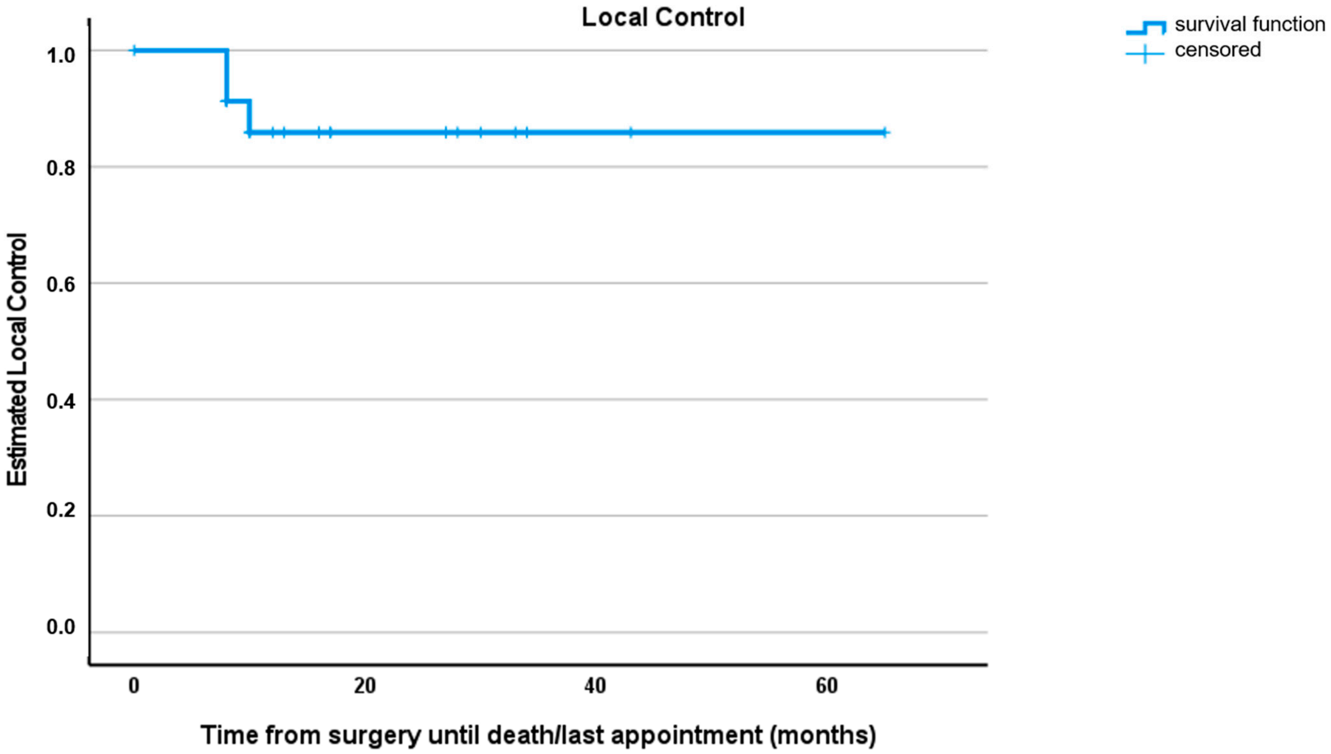

Figure S2. Kaplan-Meyer curve for local progression free survival when excluding patients receiving additional RT post IORT. Estimated LC is 85.9% (95%-CI 71.0 – 100%).
